# Glances at the Hospitals

**Published:** 1902-02-22

**Authors:** 


### GLANCES AT THE HOSPITALS.

### ST. JOSEPH'S HOSPITAL, PHILADELPHIA.

This report received by us is for the years 1899 and 1900, and during these two years 4,214 patients were treated in the house. Of these 1,500 were charity cases, and assuming that their maintenance cost $1 a day, and that the average stay in the hospital was twenty days, something like $30,000 were expended on charity work, this sum being met by the payments of private patients and by donations. The cost of administration is nothing, because no salaries are paid to the nursing sisters; and it should be particularly noted that no distinction of creed, colour, or nationality is observed. The medical and surgical tables are well arranged. There were 204 cases of enteric fever, and of these 182 recovered, and 22 died. Of 74 cases of delirium tremens, 70 recovered, and 4 died. A total number of 997 operations is recorded, but no table of the anæsthetic used is given.

### CLAYTON HOSPITAL, WAKEFIELD.

The total receipts, exclusive of legacies, amounted to £4,948, and the total ordinary expenditure to £4,014. The special expenditure was £3,048 in the purchase of land, and £2,560 for various works, furnishing new wards, etc. At the beginning of the year the hospital contained 52 patients, and 701 were admitted, making a total of 753 under treatment. Of these 509 were discharged cured, 133 relieved, 17 not relieved, 42 died, and 52 remained under treatment. The daily average number of beds occupied was 51; the highest number at one time was 67; the rate of mortality was 5·76 per cent., but if 12 patients, who died within 48 hours of admission, be deducted, the death rate would fall to 4·27 per cent. The average cost of each in-patient was £4 10s.; the average cost of each bed occupied was £65 19s. per annum. The governors note with satisfaction that the Workpeople's Fund has increased. The medical and surgical tables show the numbers cured, relieved, not relieved, and died. The operation table, on the other hand, does not give results, although 258 operations were performed. There is no information given as to the nature of the anæsthetic used.

